# Supplementary material for: Role and mechanism of NCAPD3 in promoting malignant behaviors in gastric cancer
Source: Front Pharmacol. 2024 Apr 22;15:1341039. doi: 10.3389/fphar.2024.1341039 (PMC11070777; doi:10.3389/fphar.2024.1341039)
Supplement: Supplementary file 11 [file DataSheet2.ZIP › GSEA/GO_CC/my_analysis.Gsea.1599461408869/GO_GLUTAMATERGIC_SYNAPSE.html]

Details for gene set GO\_GLUTAMATERGIC\_SYNAPSE[GSEA]

|  || Dataset | filtered\_dataset.sample\_info.cls#WT\_versus\_NCAPD3\_MUT |
| Phenotype | sample\_info.cls#WT\_versus\_NCAPD3\_MUT |
| Upregulated in class | NCAPD3\_MUT |
| GeneSet | GO\_GLUTAMATERGIC\_SYNAPSE |
| Enrichment Score (ES) | -0.3322203 |
| Normalized Enrichment Score (NES) | -1.578999 |
| Nominal p-value | 0.050480768 |
| FDR q-value | 0.13004716 |
| FWER p-Value | 0.79 |
Table: GSEA Results Summary

  

Fig 1: Enrichment plot: GO\_GLUTAMATERGIC\_SYNAPSE      
 Profile of the Running ES Score & Positions of GeneSet Members on the Rank Ordered List

  

| SYMBOL | TITLE | RANK IN GENE LIST | RANK METRIC SCORE | RUNNING ES | CORE ENRICHMENT || 1 | 613 | BCR | 180 | 0.695 | -0.0762 | No |
| 2 | 9456 | HOMER1 | 198 | 0.680 | -0.0362 | No |
| 3 | 8831 | SYNGAP1 | 236 | 0.647 | -0.0132 | No |
| 4 | 54477 | PLEKHA5 | 286 | 0.605 | -0.0021 | No |
| 5 | 200933 | FBXO45 | 456 | 0.500 | -0.0854 | No |
| 6 | 57460 | PPM1H | 502 | 0.472 | -0.0816 | No |
| 7 | 3836 | KPNA1 | 548 | 0.448 | -0.0796 | No |
| 8 | 54476 | RNF216 | 672 | 0.389 | -0.1382 | No |
| 9 | 2932 | GSK3B | 714 | 0.367 | -0.1396 | No |
| 10 | 23767 | FLRT3 | 781 | 0.333 | -0.1615 | No |
| 11 | 1605 | DAG1 | 823 | 0.304 | -0.1677 | No |
| 12 | 9181 | ARHGEF2 | 902 | -0.326 | -0.1988 | No |
| 13 | 1978 | EIF4EBP1 | 938 | -0.362 | -0.1963 | No |
| 14 | 3556 | IL1RAP | 1099 | -0.476 | -0.2749 | No |
| 15 | 7074 | TIAM1 | 1143 | -0.508 | -0.2669 | No |
| 16 | 2043 | EPHA4 | 1228 | -0.602 | -0.2811 | Yes |
| 17 | 64854 | USP46 | 1300 | -0.702 | -0.2783 | Yes |
| 18 | 3084 | NRG1 | 1320 | -0.739 | -0.2353 | Yes |
| 19 | 4734 | NEDD4 | 1369 | -0.856 | -0.2042 | Yes |
| 20 | 10397 | NDRG1 | 1380 | -0.925 | -0.1404 | Yes |
| 21 | 6711 | SPTBN1 | 1381 | -0.925 | -0.0694 | Yes |
| 22 | 493 | ATP2B4 | 1401 | -1.166 | 0.0065 | Yes |
Table: GSEA details [plain text format]

  

Fig 2: GO\_GLUTAMATERGIC\_SYNAPSE      
 Blue-Pink O' Gram in the Space of the Analyzed GeneSet

  

Fig 3: GO\_GLUTAMATERGIC\_SYNAPSE: Random ES distribution      
 Gene set null distribution of ES for **GO\_GLUTAMATERGIC\_SYNAPSE**
